# Supplementary material for: Unique amphipathic α helix drives membrane insertion and enzymatic activity of ATG3
Source: Sci Adv. 2023 Jun 23;9(25):eadh1281. doi: 10.1126/sciadv.adh1281 (PMC10289646; doi:10.1126/sciadv.adh1281)
Supplement: Supplementary file 1 — Figs. S1 to S6 Tables S1 to S2 Legends for movies S1 to S5 [file sciadv.adh1281_sm.pdf]

Science Advances
AAAS

Supplementary Materials for

Unique amphipathic α helix drives membrane insertion and enzymatic activity of ATG3

Taki Nishimura *et al.*

Corresponding author: Taki Nishimura, ntaki@m.u-tokyo.ac.jp, Roberto Covino, covino@fias.uni-frankfurt.de

Sci. Adv. **9**, eadh1281 (2023)
DOI: 10.1126/sciadv.adh1281

The PDF file includes:

Figs. S1 to S6
Tables S1 and S2
Legends for movies S1 to S5

Other Supplementary Material for this manuscript includes the following:

Movies S1 to S5

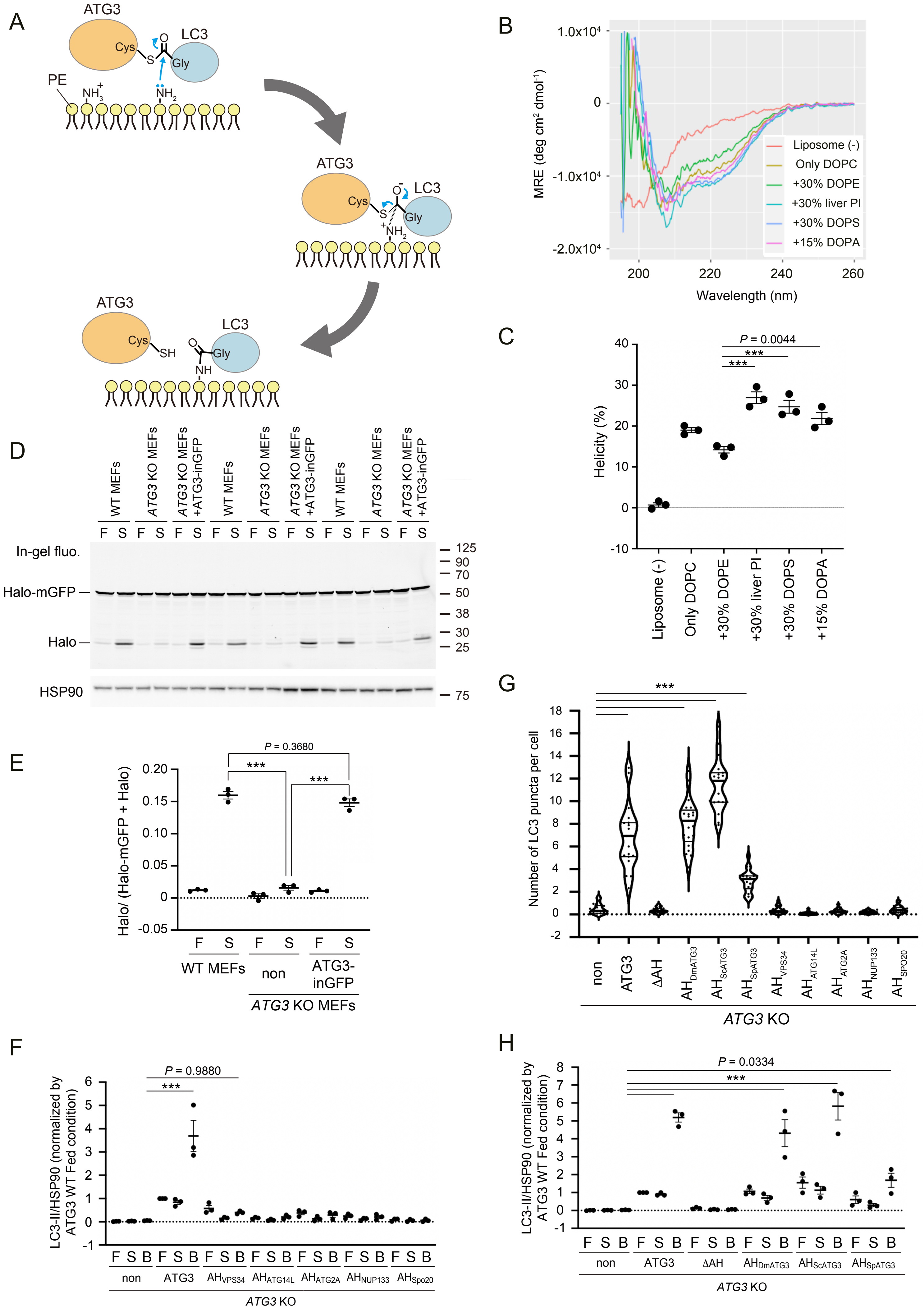
A
ATG3
LC3
Cys
Gly
PE
B
1.0x10⁴
0
-1.0x10⁴
-2.0x10⁴
MRE (deg cm² dmol⁻¹)
Wavelength (nm)
Liposome (-)
Only DOPC
+30% DOPE
+30% liver PI
+30% DOPS
+15% DOPA
C
P = 0.0044
***
Helicity (%)
D
WT MEFs
ATG3 KO MEFs
ATG3 KO MEFs +ATG3-inGFP
In-gel fluo.
Halo-mGFP
Halo
HSP90
E
P = 0.3680
***
Halo/ (Halo-mGFP + Halo)
WT MEFs
non
ATG3-inGFP
ATG3 KO MEFs
F
P = 0.9880
***
LC3-II/HSP90 (normalized by ATG3 WT Fed condition)
non
ATG3
AHVPS34
AHATG14L
AHATG2A
AHNUP133
AHSpo20
ATG3 KO
G
***
Number of LC3 puncta per cell
non
ATG3
ΔAH
AHDmATG3
AHScATG3
AHSpATG3
AHVPS34
AHATG14L
AHATG2A
AHNUP133
AHSPO20
ATG3 KO
H
P = 0.0334
***
LC3-II/HSP90 (normalized by ATG3 WT Fed condition)
non
ATG3
ΔAH
AHDmATG3
AHScATG3
AHSpATG3
ATG3 KO

Figure S1

Figure S1 related to Figure 1. ATG3-dependent LC3 conjugation reaction and quantification results of ATG3 rescue assay.

(**A**) The chemical mechanism of LC3–PE conjugation catalyzed by ATG3. (top) An ATG3~LC3 conjugate interacts with membranes. The primary amine of PE head group, which is mostly in the protonated state at physiological pH, becomes deprotonated for nucleophilic attack on the thioester bond linking ATG3 and LC3. (middle) After nucleophilic attack, the negatively charged thioester oxygen atom is formed, and it is expected to be stabilized by surrounding residues. (bottom) Simultaneously, ATG3 is dissociated from the intermediate structure and leaves the membranes, which results in the formation of an amide bond between LC3 and PE. (**B**) Far-UV CD spectra of AH_{ATG3} peptide (75 μM) in the absence or presence of sonicated liposomes (6 mM) containing increasing mol% of either DOPE, liver PI, DOPS, or DOPA. The remaining lipids in the liposome were DOPC (the concentration of which varied from 70 mol% to 100 mol% depending on the concentration of another lipid). MRE, mean residue ellipticity. (**C**) Helicity at 222 nm as determined from the spectra shown in Fig. S1B. (**D**) HaloTag processing assay. The indicated cells expressing Halo-mGFP were pulse-labeled for 30 min with 100 nM of tetramethylrhodamine (TMR)-conjugated ligand in nutrient-rich medium and then starved for 6h (S) or cultured in full media (F). Total cell lysates were subjected to immunoblotting with HSP90 antibody or in-gel fluorescence detection. (**E**) The Halo-GFP processing was calculated as the band intensity of processed Halo over that of Halo-GFP. (**F**) Band intensity quantification of LC3-II shown in Fig. 1D. All data were normalized with those of HSP90. (**G**) Quantification of the number of LC3 puncta shown in Fig. 1E. The thick and thin lines in the violin plot represent the medians and quartiles, respectively. The average number of LC3 puncta per cell was counted from randomly selected areas (n≧19). (**H**) Band intensity quantification of LC3-II shown in Fig. 1F. All data were normalized with those of HSP90. Data represent the mean ± SEM of three biological replicates (**C, E, F, H**). Differences were statistically analyzed by one-way ANOVA and Turkey multiple comparison test. ***$P < 0.001$.

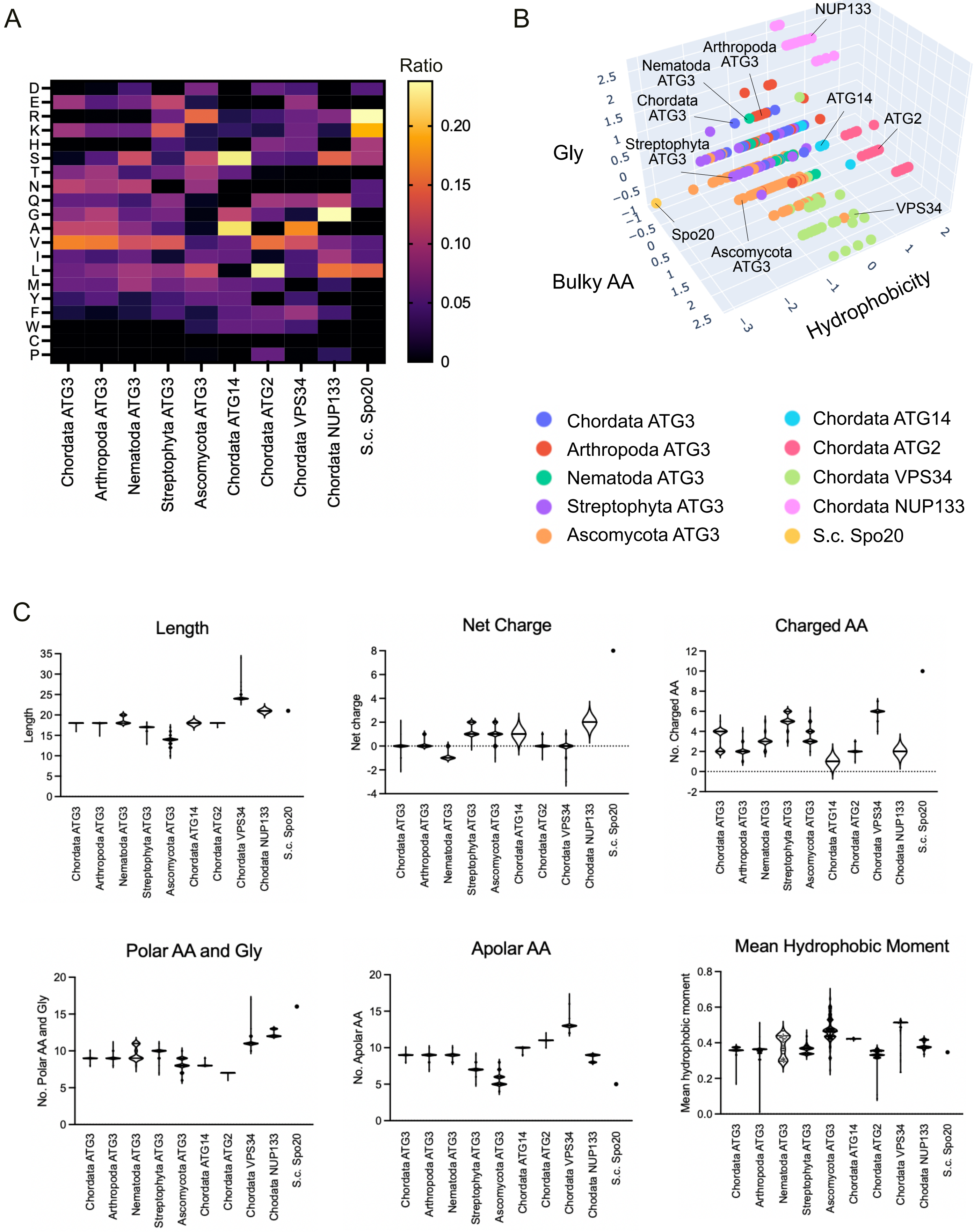
A
Ratio
D
E
R
K
H
S
T
N
Q
G
A
V
I
L
M
Y
F
W
C
P
0.20
0.15
0.10
0.05
0
Chordata ATG3
Arthropoda ATG3
Nematoda ATG3
Streptophyta ATG3
Ascomycota ATG3
Chordata ATG14
Chordata ATG2
Chordata VPS34
Chordata NUP133
S.c. Spo20
B
NUP133
Arthropoda ATG3
Nematoda ATG3
Chordata ATG3
ATG14
ATG2
Streptophyta ATG3
Gly
Bulky AA
Hydrophobicity
Spo20
Ascomycota ATG3
VPS34
Chordata ATG3
Arthropoda ATG3
Nematoda ATG3
Streptophyta ATG3
Ascomycota ATG3
Chordata ATG14
Chordata ATG2
Chordata VPS34
Chordata NUP133
S.c. Spo20
C
Length
Net Charge
Charged AA
Net charge
No. Charged AA
Polar AA and Gly
Apolar AA
Mean Hydrophobic Moment
No. Polar AA and Gly
No. Apolar AA
Mean hydrophobic moment
Chodata NUP133

Figure S2

Figure S2 related to Figure 2. Helical parameters of AHs analyzed in this study.

(**A**) Heatmap of amino acid composition of AHs. AHs derived from ATG3 proteins were categorized into five groups by phylum: Chordata, Arthropoda, Nematoda, Streptophyta, and Ascomycota. A color gradient on the right of the heatmap indicates the ratio of amino acid composition. (**B**) A 3D-plot of the number of bulky residues, glycine residues and mean hydrophobicity of AHs analyzed in this study. Each of the groups is represented by the indicated colors. (**C**) Length, net charge, the number of charged residue (Charged AA), the number of polar residue and glycine residue (Polar AA and Gly), the number of apolar residue (Apolar AA), and mean hydrophobic moment are shown. The thick and thin lines in the violin plot represent the medians and quartiles of each group, respectively.

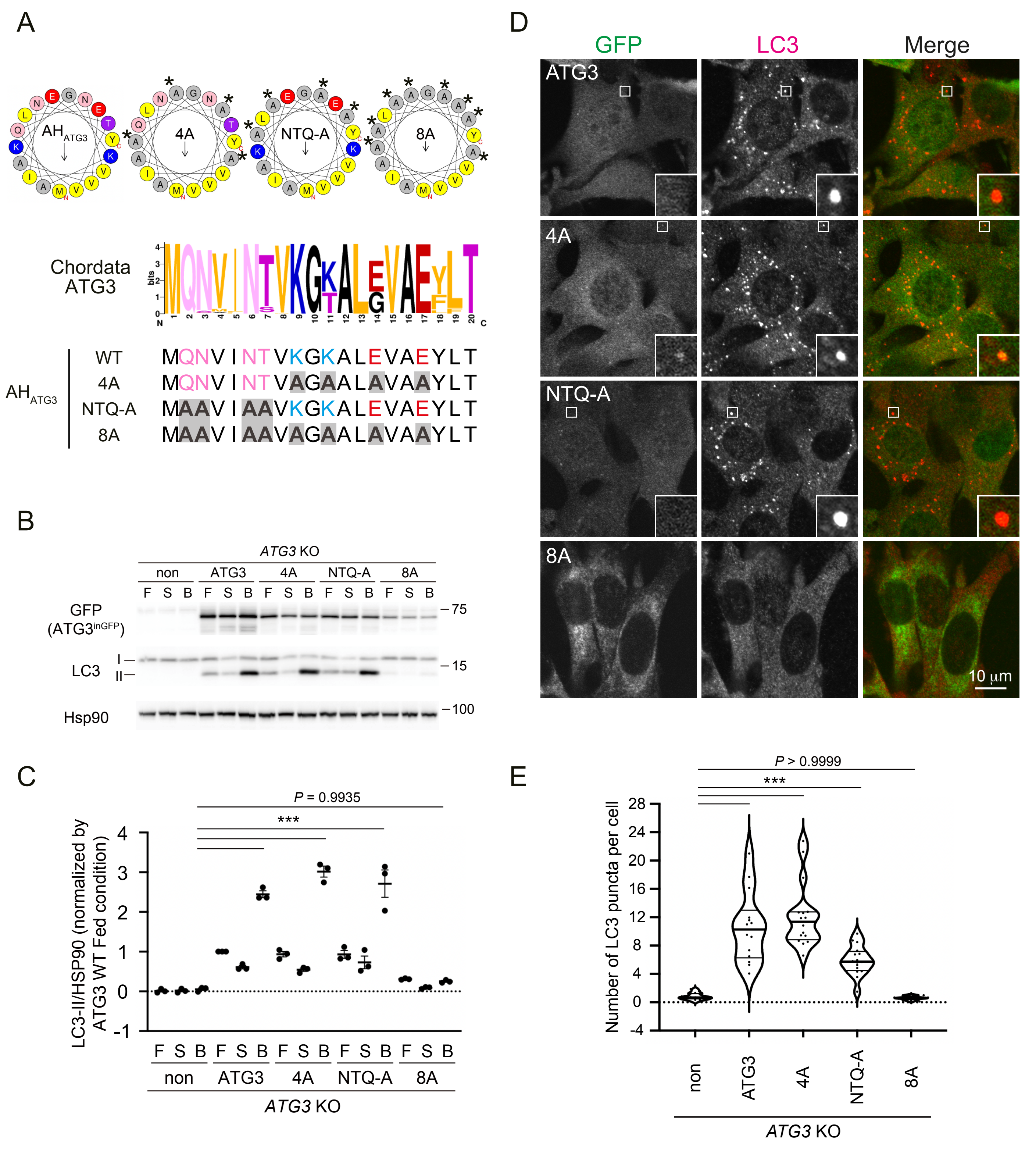
A
AH ATG3
4A
NTQ-A
8A
Chordata ATG3
WT MQNVINTVKGKALEVAEYLT
4A MQNVINTVAGAALAVAAYLT
NTQ-A MAAVIAAVKGKALEVAEYLT
8A MAAVIAAVAGAALAVAAYLT
B
ATG3 KO
non ATG3 4A NTQ-A 8A
F S B
GFP (ATG3inGFP)
LC3
Hsp90
75
15
100
C
P = 0.9935
***
LC3-II/HSP90 (normalized by ATG3 WT Fed condition)
D
GFP
LC3
Merge
ATG3
4A
NTQ-A
8A
10 μm
E
P > 0.9999
***
Number of LC3 puncta per cell
non
ATG3
4A
NTQ-A
8A
ATG3 KO

Figure S3

Figure S3 related to Figure 3. Requirement of polar residues in the hydrophilic face of AH_{ATG3}.

(**A**) (top) Helical wheel representations of AH_{ATG3} mutants containing mutations in its hydrophilic face. Asterisks indicate the position of mutations. (middle) WebLogo to represent amino acid sequence conservation among AHs of Chordata species' ATG3 proteins. (bottom) Multiple sequence alignment of AH regions of ATG3 WT and mutants. The positions of mutation are marked by gray shadow. (**B**) LC3 flux assay of *ATG3* KO cells expressing ATG3 WT or the indicated ATG3 mutants. The cells were starved for 6 h with (B) or without 100 nM Bafilomycin A_1 (S) or cultured in full media (F). Cell lysates were analyzed by immunoblotting using the indicated antibodies. (**C**) Band intensity quantification of LC3-II shown in Fig. S3B. All data were normalized with those of HSP90. Data represent the mean ± SEM of three biological replicates. (**D**) LC3 puncta formation. The cells were starved for 1 h, fixed and stained with anti-LC3 antibody. The specimens were analyzed by FV3000 confocal microscope. Scale bar, 10 μm. (**E**) Quantification of the number of LC3 puncta. The thick and thin lines in the violin plot represent the medians and quartiles, respectively. The average number of LC3 puncta per cell was counted from randomly selected areas (n≧14). Differences were statistically analyzed by one-way ANOVA and Turkey multiple comparison test. ***$P < 0.001$.

A

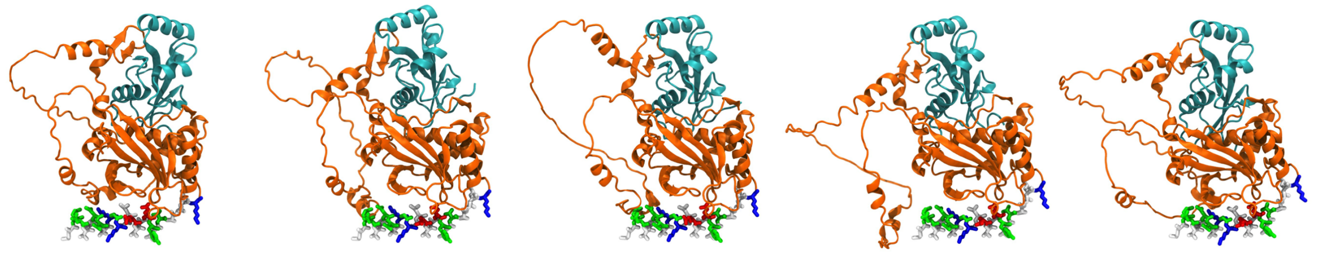

B

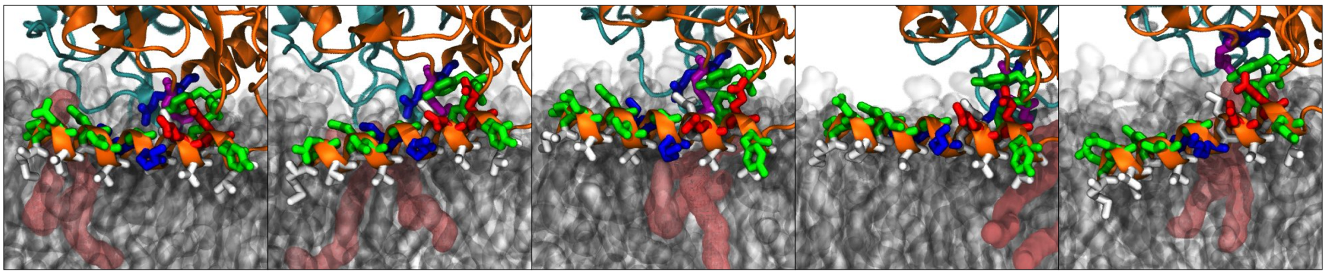

C

```
                              1         10        20        30        40        50        60        70
HUMAN_ATG3_(Chordata)         MQNVINTVKGKALEVAEYLTPVLKESKFKETGVITPEEFVAAGDHLVHHCPTWQWA.TGEELKVKAYLPTGKQFLVTKNV
DROME_Atg3_(Arthropoda)       MQSVLNTVKGTALNVAEYLTPVLKESKFRETGVLTPEEFVAAGDHLVHHCPTWQWA.AGDETKTKPYLPKDKQFLITRNV
CAEEL_Atg3_(Nematoda)         MQNLVNNLKSAALQIGETFTPVLRESKFRETGVLTPEEYVAAGDHLVHHCPTWKWAGASDPSKIRTFLPIDKQFLITRNV
ARATH_ATG3_(Streptophyta)     .MVLSQKLHEAFKGTVERITGPRTISAFKEKGVLSVSEFVLAGDNLVSKCPTWSWE.SGDASKRKPYLPSDKQFLITRNV
SCPHO_ATG3_(Ascomycota)       ...MAQRLTSAFLNWREHITPASKTSDFENTGMISPEEFVLAGDYLVSKFPTWSWE.CGD..RIRGFLPKDKQYLVTRHV
YEAST_Atg3_(Ascomycota)       ......MIRSTLSSWREYLTPITHKSTFLTTGQITPEEFVQAGDYLCHMFPTWKWNEESSDISYRDFLPKNKQFLIIRKV
                                                    *

                              80        90        100       110       120       130       140
HUMAN_ATG3_(Chordata)         PCYKRCKQM..EYSDELEAIIEEDDG......DGGWVDTYHNTGITGITEAV...KEITLENKDNIRLQDCSALCEEEED
DROME_Atg3_(Arthropoda)       PCYRRCKQM..EYVGE.ETLVEEESG......DGGWVETHQLNDDGTTQLEDKICELTMEETKEEMHTPDSDKSAPGAGG
CAEEL_Atg3_(Nematoda)         PCHKRCKQM..EYDEKLEKIINEEDGEYQTSDETGWVDTHH.........Y...EKEHEKNEEKEQSTAPPPAAPEDSD
ARATH_ATG3_(Streptophyta)     PCLRRAASVAEDYEAAGGEVLVDDED......NDGWLATHGKPKDKGKEE.D...NLPSMDALDINEKNTIQSIPTYFGG
SCPHO_ATG3_(Ascomycota)       FCVQRNINI..GVNEE....................WVDIET.DDTRNK...D...DDQDDDAISSIHSDTSDIASAERLK
YEAST_Atg3_(Ascomycota)       PCDKRAEQ.........................CVEVEG.PDVIMKGFAE...DGDEDDVLEYIGSETEHVQSTPAGG

                                  150       160       170       180                 190       200       210
HUMAN_ATG3_(Chordata)         ....EDEGEAADMEEYEESGLLETDEATLDTRKIVEACKAKTD.........AGGEDAILQTRTYDLYITYDKYYQTPRL
DROME_Atg3_(Arthropoda)       QAEDEDDDEAIDMDDFEESGMLELVDPAVATTTRKPEPEAKASPVAAASGDAEASGDSVLHTRTYDLHISYDKYYQTPRL
CAEEL_Atg3_(Nematoda)         ....DDDEEPLDLDEL....GLDDDEEDPNRFVVEKKPLAAGN.........DNSGE.VEKVRTYDLHICYDKYYQVPRL
ARATH_ATG3_(Streptophyta)     ....EEDDDIPDMEEFDEADNVVENDPAT....LQSTYLVAH.........EPDDDNILRTRTYDLSITYDKYYQTPRV
SCPHO_ATG3_(Ascomycota)       ....GQSKELSDSGPL....PLKDEEDDDQ....MVSPVIKED.........EG.........RYYDLYIVYDKYYRTPRL
YEAST_Atg3_(Ascomycota)       ....TKDSSIDDIDELIQDMEIKEEDENDD....TEEFNAKGG.........LAK.D.MAQERYYDLYIAYSTSYRVPKM
                                                     *                                                 * *   *

                              220       230       240       250       260       270
HUMAN_ATG3_(Chordata)         WLFGYDEQRQPLTVEHMYEDISQDHVKKTVTIENHPHLPPPP.MCSVHPCRHAEVMKKIIETVA................
DROME_Atg3_(Arthropoda)       WVVGYDEQRKPLTVEQMYEDVSQDHAKKTVTMESHPHLPGPN.MASVHPCRHADIMKKIIQTVE................
CAEEL_Atg3_(Nematoda)         FLMGYDENRRPLTVEQTYEDFSADHSNKTITVEAHPSV..DLTMPTVHPCKHAEMMKRLINQYA................
ARATH_ATG3_(Streptophyta)     WLTGYDESRMLLQPELVMEDVSQDHARKTVTIEDHPHLPGKH..ASVHPCRHGAVMKKIIDVLM................
SCPHO_ATG3_(Ascomycota)       FLRGWNAGGQLLTMKDIYEDVSGEHAGKTVTMEPFPHYHSHNTMASVHPCKHASVLLKLIKQHR................
YEAST_Atg3_(Ascomycota)       YIVGFNSNGSPLSPEQMFEDISADYRTKTATIEKLPFYKNSVLSVSIHPCKHANVMKILLDKVRVVRQRRRKELQEEQEL
                                                                                **

                                                280       290       300       310
HUMAN_ATG3_(Chordata)         ...........EGGGELGVHMYLLIFLKFVQAVIPTIEYDYTRHFTM.....
DROME_Atg3_(Arthropoda)       ...........EGGGQLGVHLYLIIFLKFVQTVIPTIEYDFTQNFNMS....
CAEEL_Atg3_(Nematoda)         ...........ESGKVLGVHEYLFLFLKFVQAVIPTIEYDYTRAIKL.....
ARATH_ATG3_(Streptophyta)     ...........SRGVEPEVDKYLFLFLKFMASVIPTIEYDYTMDFDLGSSST
SCPHO_ATG3_(Ascomycota)       ...........ERNDPIRVDQYMVLFLKFVSTMLPYFEIDYTIQA.......
YEAST_Atg3_(Ascomycota)       DGVGDWEDLQDDIDDSLRVDQYLIVFLKFITSVTPSIQHDYTMEGW......
```

Figure S4

Figure S4 related to Figure 4. Alpha Fold models and AH_{ATG3} dynamics in the bilayer. (**A**) Five alternative initial models of the ATG3~LC3 conjugate produced by Alpha Fold. The renders show the complex in a cartoon representation based on its secondary structure, with ATG3 in orange and LC3 in cyan. The heavy atoms of residues 1-24 (AH and linker) are also shown explicitly in a licorice representation. (**B**) Renders of representative snapshots taken every 100 ns from a trajectory illustrating the dynamics of the ATG3 AH in the bilayer. Rendering and colors as in (**A**). Lipids are showing in transparent grey. A PE lipid forming a contact with LC3 is highlighted in burgundy. (**C**) Sequence alignment of ATG3 proteins using ESPript 3. The identical and similar residues are highlighted by red and yellow boxes, respectively. The flexible region, unfolded region, and ATG12-binding region are indicated by black, cyan and magenta lines, respectively. Asterisks show the position of lipid-binding residues *in silico*.

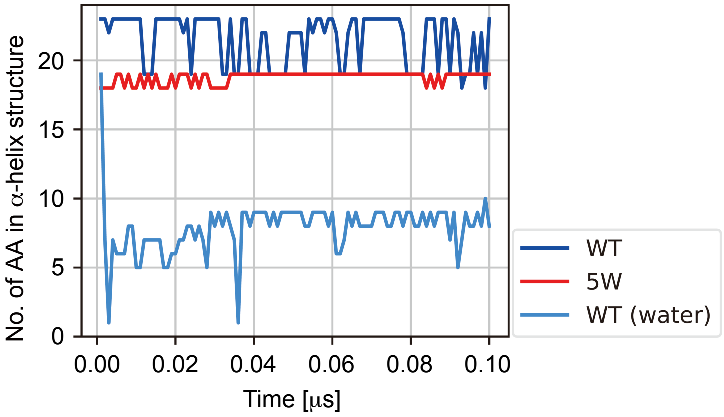
No. of AA in α-helix structure
0
5
10
15
20
0.00
0.02
0.04
0.06
0.08
0.10
Time [μs]
WT
5W
WT (water)

Figure S5

Figure S5 related to Figure 5. Stability of the AH and compactness of the ATG3~LC3 conjugate

Time series of the number of AH amino acids that are helical for WT and 5W in bilayer, and for WT in water.

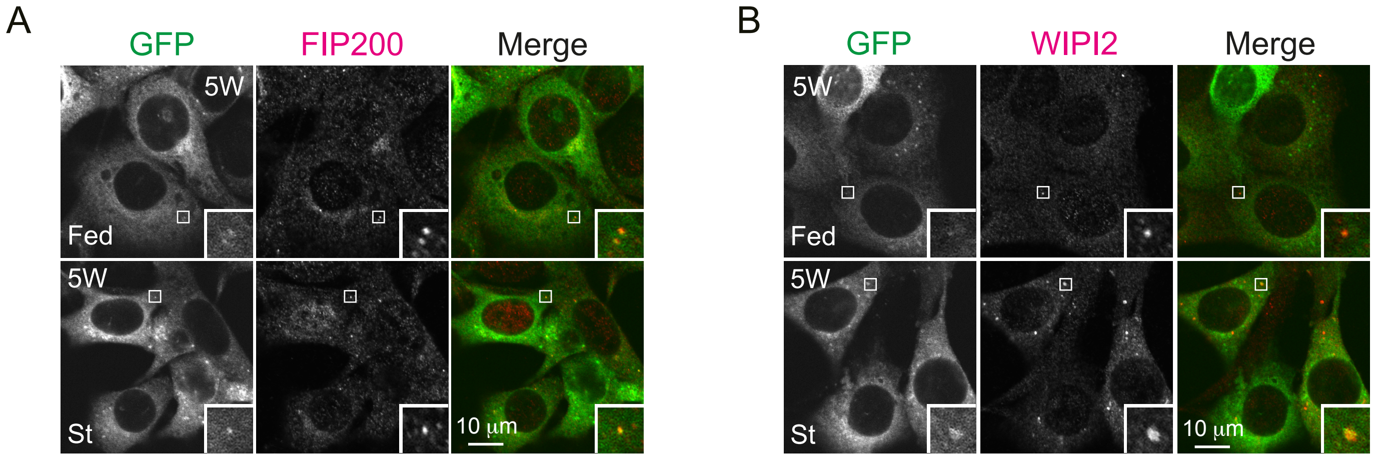
A
GFP
FIP200
Merge
5W
Fed
5W
St
10 μm
B
GFP
WIPI2
Merge
5W
Fed
5W
St
10 μm

Figure S6

Figure S6 related to Figure 6. Early ATG proteins were co-localized with 5W mutant-positive structures.

(**A, B**) *ATG3 KO* MEFs expressing 5W mutant were cultured under fed or starved condition for 1 h, fixed and stained with anti-FIP200 (**A**) or anti-WIPI2 (**B**) antibody. Note that FIP200 and WIPI2 were co-localized with 5W mutant-positive puncta under both fed and starved condition. Scale bar, 10 µm.

Supplementary Movie S1. Live-cell time-lapse images of ATG3-inGFP and Halo-ATG5 under starved condition.

Supplementary Movie S2. Live-cell time-lapse images of ATG3-inGFP and Halo-ATG5 under starved condition.

Supplementary Movie S3. Live-cell time-lapse images of ATG3ΔAH-inGFP and Halo-ATG5 under starved condition.

Supplementary Movie S4. Live-cell time-lapse images of ATG3(3W)-inGFP and Halo-ATG5 under starved condition.

Supplementary Movie S5. Live-cell time-lapse images of ATG3(5W)-inGFP and Halo-ATG5 under starved condition.

Table S1. Constructs used in this study

REAGENT or RESOURCE	SOURCE	DESCRIPTIONS
Recombinant DNA		
TN427 pMRX-IP/hATG3-inGFP<br><br>MQNVINTVKGKALEVAEYLTPVLKESKFKETGVITPE<br>EFVAAGDHLVHHCPTWQWATGEELKVKAYLPTGKQ<br>FLVTKNVPCYKRCKQMEYSDELEAIIEEDDGDGGWV<br>DTYHNTGITGITEAVKEVSKGEELFTGVVPILVELDGD<br>VNGHKFSVSGEGEGDATYGKLTLKFICTTGKLPVPW<br>PTLVTTLTYGVQCFSRYPDHMKQHDFFKSAMPEGYV<br>QERTIFFKDDGNYKTRAEVKFEGDTLVNRIELKGIDFK<br>EDGNILGHKLEYNYNSHNVYIMADKQKNGIKVNFKIR<br>HNIEDGSVQLADHYQQNTPIGDGPVLLPDNHYLSTQ<br>SALSKDPNEKRDHMVLLEFVTAAGITLGMDELYKITL<br>ENKDNIRLQDCSALCEEEEDEDEGEAADMEEYEESG<br>LLETDEATLDTRKIVEACKAKTDAGGEDAILQTRTYDL<br>YITYDKYYQTPRLWLFGYDEQRQPLTVEHMYEDISQ<br>DHVKKTVTIENHPHLPPPPMCSVHPCRHAEVMKKIIE<br>TVAEGGGELGVHMYLLIFLKFVQAVIPTIEYDYTRHFT<br>M*	This study	
TN554 pMRX-IP/AH$_{DmATG3}$-hATG3(Δ18)-inGFP<br><br>MQSVLNTVKGTALNVAEYLTPVLKESKFKETGVITPE<br>EFVAAGDHLVHHCPTWQWATGEELKVKAYLPTGKQ<br>FLVTKNVPCYKRCKQMEYSDELEAIIEEDDGDGGWV<br>DTYHNTGITGITEAVKEVSKGEELFTGVVPILVELDGD<br>VNGHKFSVSGEGEGDATYGKLTLKFICTTGKLPVPW<br>PTLVTTLTYGVQCFSRYPDHMKQHDFFKSAMPEGYV<br>QERTIFFKDDGNYKTRAEVKFEGDTLVNRIELKGIDFK<br>EDGNILGHKLEYNYNSHNVYIMADKQKNGIKVNFKIR<br>HNIEDGSVQLADHYQQNTPIGDGPVLLPDNHYLSTQ<br>SALSKDPNEKRDHMVLLEFVTAAGITLGMDELYKITL<br>ENKDNIRLQDCSALCEEEEDEDEGEAADMEEYEESG<br>LLETDEATLDTRKIVEACKAKTDAGGEDAILQTRTYDL<br>YITYDKYYQTPRLWLFGYDEQRQPLTVEHMYEDISQ<br>DHVKKTVTIENHPHLPPPPMCSVHPCRHAEVMKKIIE<br>TVAEGGGELGVHMYLLIFLKFVQAVIPTIEYDYTRHFT<br>M*	This study	Retrovirus infection
TN556 pMRX-IP/AH$_{SpATG3}$-hATG3(Δ18)-inGFP<br><br>MAQRLTSAFLNWREHLTPVLKESKFKETGVITPEEFV<br>AAGDHLVHHCPTWQWATGEELKVKAYLPTGKQFLV<br>TKNVPCYKRCKQMEYSDELEAIIEEDDGDGGWVDTY<br>HNTGITGITEAVKEVSKGEELFTGVVPILVELDGDVNG<br>HKFSVSGEGEGDATYGKLTLKFICTTGKLPVPWPTLV<br>TTLTYGVQCFSRYPDHMKQHDFFKSAMPEGYVQER<br>TIFFKDDGNYKTRAEVKFEGDTLVNRIELKGIDFKEDG<br>NILGHKLEYNYNSHNVYIMADKQKNGIKVNFKIRHNIE<br>DGSVQLADHYQQNTPIGDGPVLLPDNHYLSTQSALS<br>KDPNEKRDHMVLLEFVTAAGITLGMDELYKITLENKD<br>NIRLQDCSALCEEEEDEDEGEAADMEEYEESGLLET<br>DEATLDTRKIVEACKAKTDAGGEDAILQTRTYDLYITY<br>DKYYQTPRLWLFGYDEQRQPLTVEHMYEDISQDHV<br>KKTVTIENHPHLPPPPMCSVHPCRHAEVMKKIIETVA<br>EGGGELGVHMYLLIFLKFVQAVIPTIEYDYTRHFTM*	This study	

TN557 pMRX-IP/AH$_{ScATG3}$-hATG3(Δ18)-inGFP<br><br>MIRSTLSSWREYLTPVLKESKFKETGVITPEEFVAAG<br>DHLVHHCPTWQWATGEELKVKAYLPTGKQFLVTKN<br>VPCYKRCKQMEYSDELEAIIEEDDGDGGWVDTYHNT<br>GITGITEAVKEVSKGEELFTGVVPILVELDGDVNGHKF<br>SVSGEGEGDATYGKLTLKFICTTGKLPVPWPTLVTTL<br>TYGVQCFSRYPDHMKQHDFFKSAMPEGYVQERTIFF<br>KDDGNYKTRAEVKFEGDTLVNRIELKGIDFKEDGNIL<br>GHKLEYNYNSHNVYIMADKQKNGIKVNFKIRHNIEDG<br>SVQLADHYQQNTPIGDGPVLLPDNHYLSTQSALSKD<br>PNEKRDHMVLLEFVTAAGITLGMDELYKITLENKDNIR<br>LQDCSALCEEEEDEDEGEAADMEEYEESGLLETDEA<br>TLDTRKIVEACKAKTDAGGEDAILQTRTYDLYITYDKY<br>YQTPRLWLFGYDEQRQPLTVEHMYEDISQDHVKKTV<br>TIENHPHLPPPPMCSVHPCRHAEVMKKIIETVAEGGG<br>ELGVHMYLLIFLKFVQAVIPTIEYDYTRHFTM*	This study	Retrovirus infection
TN558 pMRX-IP/Met-AH$_{VPS34}$-hATG3(Δ18)-inGFP<br><br>MDESVHALFAAVVEQIHKFAQYWRKLTPVLKESKFK<br>ETGVITPEEFVAAGDHLVHHCPTWQWATGEELKVKA<br>YLPTGKQFLVTKNVPCYKRCKQMEYSDELEAIIEEDD<br>GDGGWVDTYHNTGITGITEAVKEVSKGEELFTGVVPI<br>LVELDGDVNGHKFSVSGEGEGDATYGKLTLKFICTT<br>GKLPVPWPTLVTTLTYGVQCFSRYPDHMKQHDFFKS<br>AMPEGYVQERTIFFKDDGNYKTRAEVKFEGDTLVNRI<br>ELKGIDFKEDGNILGHKLEYNYNSHNVYIMADKQKNG<br>IKVNFKIRHNIEDGSVQLADHYQQNTPIGDGPVLLPD<br>NHYLSTQSALSKDPNEKRDHMVLLEFVTAAGITLGM<br>DELYKITLENKDNIRLQDCSALCEEEEDEDEGEAADM<br>EEYEESGLLETDEATLDTRKIVEACKAKTDAGGEDAI<br>LQTRTYDLYITYDKYYQTPRLWLFGYDEQRQPLTVE<br>HMYEDISQDHVKKTVTIENHPHLPPPPMCSVHPCRH<br>AEVMKKIIETVAEGGGELGVHMYLLIFLKFVQAVIPTIE<br>YDYTRHFTM*	This study	
TN559 pMRX-IP/Met-AH$_{NUP133}$-hATG3(Δ18)-inGFP<br><br>MLPQGQGMLSGIGRKVSSLFGILTPVLKESKFKETGV<br>ITPEEFVAAGDHLVHHCPTWQWATGEELKVKAYLPT<br>GKQFLVTKNVPCYKRCKQMEYSDELEAIIEEDDGDG<br>GWVDTYHNTGITGITEAVKEVSKGEELFTGVVPILVEL<br>DGDVNGHKFSVSGEGEGDATYGKLTLKFICTTGKLP<br>VPWPTLVTTLTYGVQCFSRYPDHMKQHDFFKSAMP<br>EGYVQERTIFFKDDGNYKTRAEVKFEGDTLVNRIELK<br>GIDFKEDGNILGHKLEYNYNSHNVYIMADKQKNGIKV<br>NFKIRHNIEDGSVQLADHYQQNTPIGDGPVLLPDNHY<br>LSTQSALSKDPNEKRDHMVLLEFVTAAGITLGMDELY<br>KITLENKDNIRLQDCSALCEEEEDEDEGEAADMEEYE<br>ESGLLETDEATLDTRKIVEACKAKTDAGGEDAILQTR<br>TYDLYITYDKYYQTPRLWLFGYDEQRQPLTVEHMYE<br>DISQDHVKKTVTIENHPHLPPPPMCSVHPCRHAEVM<br>KKIIETVAEGGGELGVHMYLLIFLKFVQAVIPTIEYDYT<br>RHFTM*	This study	

TN560 pMRX-IP/Met-AH$_{SPO20}$-hATG3(Δ18)-inGFP<br><br>MSRRRDRLHVKLKSLRNKIHKQLTPVLKESKFKETGV<br>ITPEEFVAAGDHLVHHCPTWQWATGEELKVKAYLPT<br>GKQFLVTKNVPCYKRCKQMEYSDELEAIIEEDDGDG<br>GWVDTYHNTGITGITEAVKEVSKGEELFTGVVPILVEL<br>DGDVNGHKFSVSGEGEGDATYGKLTLKFICTTGKLP<br>VPWPTLVTTLTYGVQCFSRYPDHMKQHDFFKSAMP<br>EGYVQERTIFFKDDGNYKTRAEVKFEGDTLVNRIELK<br>GIDFKEDGNILGHKLEYNYNSHNVYIMADKQKNGIKV<br>NFKIRHNIEDGSVQLADHYQQNTPIGDGPVLLPDNHY<br>LSTQSALSKDPNEKRDHMVLLEFVTAAGITLGMDELY<br>KITLENKDNIRLQDCSALCEEEEDEDEGEAADMEEYE<br>ESGLLETDEATLDTRKIVEACKAKTDAGGEDAILQTR<br>TYDLYITYDKYYQTPRLWLFGYDEQRQPLTVEHMYE<br>DISQDHVKKTVTIENHPHLPPPPMCSVHPCRHAEVM<br>KKIIETVAEGGGELGVHMYLLIFLKFVQAVIPTIEYDYT<br>RHFTM*	This study	
TN561 pMRX-IP/Met-AH$_{ATG14L}$-hATG3(Δ18)-inGFP<br><br>MGGMISSAAASVTSWFKAYLTPVLKESKFKETGVITP<br>EEFVAAGDHLVHHCPTWQWATGEELKVKAYLPTGK<br>QFLVTKNVPCYKRCKQMEYSDELEAIIEEDDGDGGW<br>VDTYHNTGITGITEAVKEVSKGEELFTGVVPILVELDG<br>DVNGHKFSVSGEGEGDATYGKLTLKFICTTGKLPVP<br>WPTLVTTLTYGVQCFSRYPDHMKQHDFFKSAMPEG<br>YVQERTIFFKDDGNYKTRAEVKFEGDTLVNRIELKGI<br>DFKEDGNILGHKLEYNYNSHNVYIMADKQKNGIKVNF<br>KIRHNIEDGSVQLADHYQQNTPIGDGPVLLPDNHYLS<br>TQSALSKDPNEKRDHMVLLEFVTAAGITLGMDELYKI<br>TLENKDNIRLQDCSALCEEEEDEDEGEAADMEEYEE<br>SGLLETDEATLDTRKIVEACKAKTDAGGEDAILQTRT<br>YDLYITYDKYYQTPRLWLFGYDEQRQPLTVEHMYEDI<br>SQDHVKKTVTIENHPHLPPPPMCSVHPCRHAEVMKK<br>IIETVAEGGGELGVHMYLLIFLKFVQAVIPTIEYDYTRH<br>FTM*	This study	Retrovirus infection
TN562 pMRX-IP/Met-AH$_{ATG2A}$-hATG3(Δ18)-inGFP<br><br>MPMHSVVQLFQGFRDLLWLLTPVLKESKFKETGVITP<br>EEFVAAGDHLVHHCPTWQWATGEELKVKAYLPTGK<br>QFLVTKNVPCYKRCKQMEYSDELEAIIEEDDGDGGW<br>VDTYHNTGITGITEAVKEVSKGEELFTGVVPILVELDG<br>DVNGHKFSVSGEGEGDATYGKLTLKFICTTGKLPVP<br>WPTLVTTLTYGVQCFSRYPDHMKQHDFFKSAMPEG<br>YVQERTIFFKDDGNYKTRAEVKFEGDTLVNRIELKGI<br>DFKEDGNILGHKLEYNYNSHNVYIMADKQKNGIKVNF<br>KIRHNIEDGSVQLADHYQQNTPIGDGPVLLPDNHYLS<br>TQSALSKDPNEKRDHMVLLEFVTAAGITLGMDELYKI<br>TLENKDNIRLQDCSALCEEEEDEDEGEAADMEEYEE<br>SGLLETDEATLDTRKIVEACKAKTDAGGEDAILQTRT<br>YDLYITYDKYYQTPRLWLFGYDEQRQPLTVEHMYEDI<br>SQDHVKKTVTIENHPHLPPPPMCSVHPCRHAEVMKK<br>IIETVAEGGGELGVHMYLLIFLKFVQAVIPTIEYDYTRH<br>FTM*	This study	

TN583 pMRX-IP/hATG3(3W: I5W/V8W/V15W)-inGFP<br><br>MQNVWNTWKGKALEWAEYLTPVLKESKFKETGVITP<br>EEFVAAGDHLVHHCPTWQWATGEELKVKAYLPTGK<br>QFLVTKNVPCYKRCKQMEYSDELEAIIEEDDGDGGW<br>VDTYHNTGITGITEAVKEVSKGEELFTGVVPILVELDG<br>DVNGHKFSVSGEGEGDATYGKLTLKFICTTGKLPVP<br>WPTLVTTLTYGVQCFSRYPDHMKQHDFFKSAMPEG<br>YVQERTIFFKDDGNYKTRAEVKFEGDTLVNRIELKGI<br>DFKEDGNILGHKLEYNYNSHNVYIMADKQKNGIKVNF<br>KIRHNIEDGSVQLADHYQQNTPIGDGPVLLPDNHYLS<br>TQSALSKDPNEKRDHMVLLEFVTAAGITLGMDELYKI<br>TLENKDNIRLQDCSALCEEEEDEDEGEAADMEEYEE<br>SGLLETDEATLDTRKIVEACKAKTDAGGEDAILQTRT<br>YDLYITYDKYYQTPRLWLFGYDEQRQPLTVEHMYEDI<br>SQDHVKKTVTIENHPHLPPPPMCSVHPCRHAEVMKK<br>IIETVAEGGGELGVHMYLLIFLKFVQAVIPTIEYDYTRH<br>FTM*	This study	
TN586 pMRX-IP/hATG3(4A: K9A/K11A/E14A/E17A)-inGFP<br><br>MQNVINTVAGAALAVAAYLTPVLKESKFKETGVITPE<br>EFVAAGDHLVHHCPTWQWATGEELKVKAYLPTGKQ<br>FLVTKNVPCYKRCKQMEYSDELEAIIEEDDGDGGWV<br>DTYHNTGITGITEAVKEVSKGEELFTGVVPILVELDGD<br>VNGHKFSVSGEGEGDATYGKLTLKFICTTGKLPVPW<br>PTLVTTLTYGVQCFSRYPDHMKQHDFFKSAMPEGYV<br>QERTIFFKDDGNYKTRAEVKFEGDTLVNRIELKGIDFK<br>EDGNILGHKLEYNYNSHNVYIMADKQKNGIKVNFKIR<br>HNIEDGSVQLADHYQQNTPIGDGPVLLPDNHYLSTQ<br>SALSKDPNEKRDHMVLLEFVTAAGITLGMDELYKITL<br>ENKDNIRLQDCSALCEEEEDEDEGEAADMEEYEESG<br>LLETDEATLDTRKIVEACKAKTDAGGEDAILQTRTYDL<br>YITYDKYYQTPRLWLFGYDEQRQPLTVEHMYEDISQ<br>DHVKKTVTIENHPHLPPPPMCSVHPCRHAEVMKKIIE<br>TVAEGGGELGVHMYLLIFLKFVQAVIPTIEYDYTRHFT<br>M*	This study	Retrovirus infection
TN587 pMRX-IP/Met-hATG3(Δ18)-inGFP<br><br>MLTPVLKESKFKETGVITPEEFVAAGDHLVHHCPTW<br>QWATGEELKVKAYLPTGKQFLVTKNVPCYKRCKQM<br>EYSDELEAIIEEDDGDGGWVDTYHNTGITGITEAVKE<br>VSKGEELFTGVVPILVELDGDVNGHKFSVSGEGEGD<br>ATYGKLTLKFICTTGKLPVPWPTLVTTLTYGVQCFSR<br>YPDHMKQHDFFKSAMPEGYVQERTIFFKDDGNYKT<br>RAEVKFEGDTLVNRIELKGIDFKEDGNILGHKLEYNY<br>NSHNVYIMADKQKNGIKVNFKIRHNIEDGSVQLADHY<br>QQNTPIGDGPVLLPDNHYLSTQSALSKDPNEKRDHM<br>VLLEFVTAAGITLGMDELYKITLENKDNIRLQDCSALC<br>EEEEDEDEGEAADMEEYEESGLLETDEATLDTRKIVE<br>ACKAKTDAGGEDAILQTRTYDLYITYDKYYQTPRLWL<br>FGYDEQRQPLTVEHMYEDISQDHVKKTVTIENHPHL<br>PPPPMCSVHPCRHAEVMKKIIETVAEGGGELGVHMY<br>LLIFLKFVQAVIPTIEYDYTRHFTM*	This study	

TN588 pMRX-IP/hATG3(V8K)-inGFP<br><br>MQNVINTKKGKALEVAEYLTPVLKESKFKETGVITPE<br>EFVAAGDHLVHHCPTWQWATGEELKVKAYLPTGKQ<br>FLVTKNVPCYKRCKQMEYSDELEAIIEEDDGDGGWV<br>DTYHNTGITGITEAVKEVSKGEELFTGVVPILVELDGD<br>VNGHKFSVSGEGEGDATYGKLTLKFICTTGKLPVPW<br>PTLVTTLTYGVQCFSRYPDHMKQHDFFKSAMPEGYV<br>QERTIFFKDDGNYKTRAEVKFEGDTLVNRIELKGIDFK<br>EDGNILGHKLEYNYNSHNVYIMADKQKNGIKVNFKIR<br>HNIEDGSVQLADHYQQNTPIGDGPVLLPDNHYLSTQ<br>SALSKDPNEKRDHMVLLEFVTAAGITLGMDELYKITL<br>ENKDNIRLQDCSALCEEEEDEDEGEAADMEEYEESG<br>LLETDEATLDTRKIVEACKAKTDAGGEDAILQTRTYDL<br>YITYDKYYQTPRLWLFGYDEQRQPLTVEHMYEDISQ<br>DHVKKTVTIENHPHLPPPPMCSVHPCRHAEVMKKIIE<br>TVAEGGGELGVHMYLLIFLKFVQAVIPTIEYDYTRHFT<br>M*	This study	
TN595 pMRX-IP/hATG3(NTQ-A: Q2A/N3A/N6A/T7A)-inGFP<br><br>MAAVIAAVKGKALEVAEYLTPVLKESKFKETGVITPEE<br>FVAAGDHLVHHCPTWQWATGEELKVKAYLPTGKQF<br>LVTKNVPCYKRCKQMEYSDELEAIIEEDDGDGGWVD<br>TYHNTGITGITEAVKEVSKGEELFTGVVPILVELDGDV<br>NGHKFSVSGEGEGDATYGKLTLKFICTTGKLPVPWP<br>TLVTTLTYGVQCFSRYPDHMKQHDFFKSAMPEGYV<br>QERTIFFKDDGNYKTRAEVKFEGDTLVNRIELKGIDFK<br>EDGNILGHKLEYNYNSHNVYIMADKQKNGIKVNFKIR<br>HNIEDGSVQLADHYQQNTPIGDGPVLLPDNHYLSTQ<br>SALSKDPNEKRDHMVLLEFVTAAGITLGMDELYKITL<br>ENKDNIRLQDCSALCEEEEDEDEGEAADMEEYEESG<br>LLETDEATLDTRKIVEACKAKTDAGGEDAILQTRTYDL<br>YITYDKYYQTPRLWLFGYDEQRQPLTVEHMYEDISQ<br>DHVKKTVTIENHPHLPPPPMCSVHPCRHAEVMKKIIE<br>TVAEGGGELGVHMYLLIFLKFVQAVIPTIEYDYTRHFT<br>M*	This study	Retrovirus infection
TN596 pMRX-IP/hATG3(2W: V4W/V8W)-inGFP<br><br>MQNWINTWKGKALEVAEYLTPVLKESKFKETGVITPE<br>EFVAAGDHLVHHCPTWQWATGEELKVKAYLPTGKQ<br>FLVTKNVPCYKRCKQMEYSDELEAIIEEDDGDGGWV<br>DTYHNTGITGITEAVKEVSKGEELFTGVVPILVELDGD<br>VNGHKFSVSGEGEGDATYGKLTLKFICTTGKLPVPW<br>PTLVTTLTYGVQCFSRYPDHMKQHDFFKSAMPEGYV<br>QERTIFFKDDGNYKTRAEVKFEGDTLVNRIELKGIDFK<br>EDGNILGHKLEYNYNSHNVYIMADKQKNGIKVNFKIR<br>HNIEDGSVQLADHYQQNTPIGDGPVLLPDNHYLSTQ<br>SALSKDPNEKRDHMVLLEFVTAAGITLGMDELYKITL<br>ENKDNIRLQDCSALCEEEEDEDEGEAADMEEYEESG<br>LLETDEATLDTRKIVEACKAKTDAGGEDAILQTRTYDL<br>YITYDKYYQTPRLWLFGYDEQRQPLTVEHMYEDISQ<br>DHVKKTVTIENHPHLPPPPMCSVHPCRHAEVMKKIIE<br>TVAEGGGELGVHMYLLIFLKFVQAVIPTIEYDYTRHFT<br>M*	This study	

TN603 pMRX-IP/hATG3(5W: V4W/V8W/A12W/V15W/A16W)-inGFP<br><br>MQNWINTWKGKWLEWWEYLTPVLKESKFKETGVIT PEEFVAAGDHLVHHCPTWQWATGEELKVKAYLPTG KQFLVTKNVPCYKRCKQMEYSDELEAIIEEDDGDGG WVDTYHNTGITGITEAVKEVSKGEELFTGVVPILVELD GDVNGHKFSVSGEGEGDATYGKLTLKFICTTGKLPV PWPTLVTTLTYGVQCFSRYPDHMKQHDFFKSAMPE GYVQERTIFFKDDGNYKTRAEVKFEGDTLVNRIELKG IDFKEDGNILGHKLEYNYNSHNVYIMADKQKNGIKVN FKIRHNIEDGSVQLADHYQQNTPIGDGPVLLPDNHYL STQSALSKDPNEKRDHMVLLEFVTAAGITLGMDELYK ITLENKDNIRLQDCSALCEEEEDEDEGEAADMEEYEE SGLLETDEATLDTRKIVEACKAKTDAGGEDAILQTRT YDLYITYDKYYQTPRLWLFGYDEQRQPLTVEHMYEDI SQDHVKKTVTIENHPHLPPPPMCSVHPCRHAEVMKK IIETVAEGGGELGVHMYLLIFLKFVQAVIPTIEYDYTRH FTM*	This study	Retrovirus infection
TN614 pMRX-IP/Met-AH$_{ATG2A\text{-}m1}$-hATG3(Δ18)-inGFP<br><br>MPMHSVVQLVQGVRDLLVLLTPVLKESKFKETGVITP EEFVAAGDHLVHHCPTWQWATGEELKVKAYLPTGK QFLVTKNVPCYKRCKQMEYSDELEAIIEEDDGDGGW VDTYHNTGITGITEAVKEVSKGEELFTGVVPILVELDG DVNGHKFSVSGEGEGDATYGKLTLKFICTTGKLPVP WPTLVTTLTYGVQCFSRYPDHMKQHDFFKSAMPEG YVQERTIFFKDDGNYKTRAEVKFEGDTLVNRIELKGI DFKEDGNILGHKLEYNYNSHNVYIMADKQKNGIKVNF KIRHNIEDGSVQLADHYQQNTPIGDGPVLLPDNHYLS TQSALSKDPNEKRDHMVLLEFVTAAGITLGMDELYKI TLENKDNIRLQDCSALCEEEEDEDEGEAADMEEYEE SGLLETDEATLDTRKIVEACKAKTDAGGEDAILQTRT YDLYITYDKYYQTPRLWLFGYDEQRQPLTVEHMYEDI SQDHVKKTVTIENHPHLPPPPMCSVHPCRHAEVMKK IIETVAEGGGELGVHMYLLIFLKFVQAVIPTIEYDYTRH FTM*	This study	
TN617 pMRX-IP/hATG3(8A: Q2A/N3A/N6A/T7A/K9A/K11A/E14A/E17A)-inGFP<br><br>MAAVIAAVAGAALAVAAYLTPVLKESKFKETGVITPEE FVAAGDHLVHHCPTWQWATGEELKVKAYLPTGKQF LVTKNVPCYKRCKQMEYSDELEAIIEEDDGDGGWVD TYHNTGITGITEAVKEVSKGEELFTGVVPILVELDGDV NGHKFSVSGEGEGDATYGKLTLKFICTTGKLPVPWP TLVTTLTYGVQCFSRYPDHMKQHDFFKSAMPEGYV QERTIFFKDDGNYKTRAEVKFEGDTLVNRIELKGIDFK EDGNILGHKLEYNYNSHNVYIMADKQKNGIKVNFKIR HNIEDGSVQLADHYQQNTPIGDGPVLLPDNHYLSTQ SALSKDPNEKRDHMVLLEFVTAAGITLGMDELYKITL ENKDNIRLQDCSALCEEEEDEDEGEAADMEEYEESG LLETDEATLDTRKIVEACKAKTDAGGEDAILQTRTYDL YITYDKYYQTPRLWLFGYDEQRQPLTVEHMYEDISQ DHVKKTVTIENHPHLPPPPMCSVHPCRHAEVMKKIIE TVAEGGGELGVHMYLLIFLKFVQAVIPTIEYDYTRHFT M*	This study	

TN834 pMRX-IPU/HaloTag-mATG5<br><br>MEIGTGFPFDPHYVEVLGERMHYVDVGPRDGTPVLF<br>LHGNPTSSYVWRNIIPHVAPTHRCIAPDLIGMGKSDK<br>PDLGYFFDDHVRFMDAFIEALGLEEVVLVIHDWGSAL<br>GFHWAKRNPERVKGIAFMEFIRPIPTWDEWPEFARE<br>TFQAFRTTDVGRKLIIDQNVFIEGTLPMGVVRPLTEVE<br>MDHYREPFLNPVDREPLWRFPNELPIAGEPANIVALV<br>EEYMDWLHQSPVPKLLFWGTPGVLIPPAEAARLAKS<br>LPNCKAVDIGPGLNLLQEDNPDLIGSEIARWLSTLEIS<br>GGSGSGSGNSMTDDKDVLRDVWFGRIPTCFTLYQD<br>EITEREAEPYYLLLPRVSYLTLVTDKVKKHFQKVMRQ<br>EDVSEIWFEYEGTPLKWHYPIGLLFDLLASSSALPWN<br>ITVHFKSFPEKDLLHCPSKDAVEAHFMSCMKEADALK<br>HKSQVINEMQKKDHKQLWMGLQNDRFDQFWAINRK<br>LMEYPPEENGFRYIPFRIYQTTTERPFIQKLFRPVAAD<br>GQLHTLGDLLREVCPSAVAPEDGEKRSQVMIHGIEP<br>MLETPLQWLSEHLSYPDNFLHISIVPQPTD*	This study	Retrovirus infection
TN928 pMRX-IBU/HaloTag-mGFP	Yim et al, 2022 (*32*)	

Table S2. Primers used in this study

Primer ID	Plasmid	Sequence
530_ATG3_fw	TN427	5'-agctagttaattaaggcggccgcATGCAGAATGTGATTAATACTGTG-3'
608_ATG3-GFP1_fw1		5'-ACGGAAGCCGTTAAAGAGGTGAGCAAGGGCGAGGAG-3'
609_ATG3-GFP1_rv1		5'-CTCCTCGCCCTTGCTCACCTCTTTAACGGCTTCCGT-3'
610_ATG3-GFP1_fw2		5'-ATGGACGAGCTGTACAAGATCACACTGGAAAATAAG-3'
611_ATG3-GFP1_rv2		5'-CTTATTTTCCAGTGTGATCTTGTACAGCTCGTCCAT-3'
531_ATG3_rv		5'-ggcggaatttacgtagcggccgcTTACATTGTGAAGTG-3'
590_DroAH_NotI_fw	TN554	5'-agctagttaattaaggcggccgcatgcagagcgtgctgaacaccgtgaaaggcaccgc-3'
591_DroAH_rv		5'-GAGGACCGGGGTCAGatattccgccacgttcagcgcggtgcctttcacggtgttcagc-3'
592_DroAH_fw2		5'-aacgtggcggaatatCTGACCCCGGTCCTC-3'
599_SpAH_NotI_fw	TN556	5'-agctagttaattaaggcggccgcatggcgcagcgcctgaccagcgcgtttc-3'
600_SpAH_rv		5'-GAGGACCGGGGTCAGatgttcgcgccagttcagaaacgcgctggtcaggc-3'
601_SpAH_fw2		5'-actggcgcgaacatCTGACCCCGGTCCTC-3'
602_ScAH_NotI_fw	TN557	5'-agctagttaattaaggcggccgcatgattcgcagcaccctgagcag-3'
603_ScAH_rv		5'-GAGGACCGGGGTCAGatattcgcgccagctgctcagggtgctgcg-3'
604_ScAH_fw2		5'-tggcgcgaatatCTGACCCCGGTCCTC-3'
35_VPS34_fw	TN558	5'-agctagttaattaaggcggccgcATGgatgagagtgtccatg-3'
37_VPS34-ATG3_rv		5'-TGAGGACCGGGGTCAGttttctccagtactgg-3'
36_VPS34-ATG3_fw		5'-ccagtactggagaaaaCTGACCCCGGTCCTCA-3'
38_NUP133_fw	TN559	5'-agctagttaattaaggcggccgcATGctgcctcaggggcaagg-3'
40_NUP133-ATG3_rv		5'-CTTGAGGACCGGGGTCAGaattccaaaaagagaaga-3'
39_NUP133-ATG3_fw		5'-tcttctctttttggaattCTGACCCCGGTCCTCAAG-3'
41_Spo20_fw	TN560	5'-agctagttaattaaggcggccgcATGagcagaagacgtga-3'

43_Spo20-ATG3_rv	TN560	5’-CTTGAGGACCGGGGTCAGttgtttgtggattttatt-3’
42_Spo20-ATG3_fw		5’-aataaaatccacaaacaaCTGACCCCGGTCCTCAAG-3’
542_14AH-ATG3_fw	TN561	5’-agctagttaattaaggcggccgcATGggtgggatgatct-3’
543_14AH-ATG3_rv		5’-TGAGGACCGGGGTCAGgtaagctttaaacc-3’
544_14AH-ATG3_fw2		5’-ggtttaaagcttacCTGACCCCGGTCCTCA-3’
548_2AH-ATG3_fw	TN562	5’-agctagttaattaaggcggccgcatgcccatgcactcggttg-3’
549_2AH-ATG3_rv		5’-GAGGACCGGGGTCAGcagccacagcaggtc-3’
550_2AH-ATG3_fw2		5’-gacctgctgtggctgCTGACCCCGGTCCTC-3’
534_ATG3-3W_fw	TN583	5’-agctagttaattaaggcggccgcATGCAGAATGTGtggAATACTtggAAGGGAAAG-3’
535_ATG3-3W_rv		5’-GAGGACCGGGGTCAGGTACTCAGCccaTTCCAGTGCCTTTCCCTTccaAGTAT-3’
536_ATG3_seq1_fw		5’-TGACCCCGGTCCTCAAGGAATCAAAGTTTAAGG-3’
540_ATG3-4A_fw	TN586	5’-agctagttaattaaggcggccgcATGCAGAATGTGATTAATACTGTGgcGGGAgcGG-3’
541_ATG3-4A_rv		5’-TGAGGACCGGGGTCAGGTACgcAGCCACTgcCAGTGCCgcTCCCgcCACAGTATTAAT-3’
532_ATG3 d18_fw	TN587	5’-agctagttaattaaggcggccgcATGCTGACCCCGGTC-3’
533_V8K_fw	TN588	5’-tagctagttaattaaggcggccgcATGCAGAATGTGATTAATACTaaGAAGGGAAAGG-3’
102_NTQ-A_fw	TN595	5’-agctagttaattaaggcggccgcATGgcGgcTGTGATTgcTgCTGTGAAGGGAAAGG-3’

103_NTQ-A_rv	TN595	5'-GAGGACCGGGGTCAGGTACTCAGCCACTTCCAGTGCCTTTCCCTTCACAGcAgc-3'
104_5W mut_fw	TN596<br>TN603	5'-agctagttaattaaggcggccgcATGCAGAATtgGATTAATACTtgGAAGGGAAAG-3'
105_5W mut_rv		5'-GAGGACCGGGGTCAGGTACTCccaCcaTTCCAGccaCTTTCCCTTCcaAGTAT-3'
133_2A-AH_mu1_fw	TN614	5'-agctagttaattaaggcggccgcatgcccatgcactcggttgtccagctcGtccaa-3'
134_2A-AH_mu1_rv		5'-GAGGACCGGGGTCAGcagcACcagcaggtcccggaCcccttggaCgagctggacaac-3'
135_2A-AH_mu1_fw2		5'-gacctgctgGTgctgCTGACCCCGGTCCTCA-3'
140_8A mut_fw	TN617	5'-agctagttaattaaggcggccgcATGgcGgcTGTGATTgcTgCTGTGgcGGGAgcG-3'
141_8A mut_rv		5'-GAGGACCGGGGTCAGGTACgCAGCCACTgCCAGTGCCgcTCCCgcCACAGcAgc-3'
142_8A mut_fw2		5'-GcAGTGGCTGcGTACCTGACCCCGGTCCTCA-3'
252_mATG5_fw	TN834	5'-aggctcgGGATCCggGAATTCgatgacagatgacaaa-3'
253_mATG5_rv		5'-gcggaatttacgtaGCGGCCGCtcaatctgttggctgggg-3'
